# Prescription Patterns of Chinese Herbal Products for Osteoporosis in Taiwan: A Population-Based Study

**DOI:** 10.1155/2012/752837

**Published:** 2012-10-10

**Authors:** Wei-Tai Shih, Yao-Hsu Yang, Pau-Chung Chen

**Affiliations:** ^1^Department for Traditional Chinese Medicine, Chang Gung Memorial Hospital, Chia-Yi 61363, Taiwan; ^2^Institute of Occupational Medicine and Industrial Hygiene, National Taiwan University College of Public Health, Taipei 10055, Taiwan; ^3^Department of Public Health, National Taiwan University College of Public Health, Taipei 10055, Taiwan; ^4^Department of Environmental and Occupational Medicine, National Taiwan University Hospital and National Taiwan University College of Medicine, Taipei 10055, Taiwan

## Abstract

*Background*. Traditional Chinese medicine (TCM) includes Chinese herbal products (CHPs), acupuncture, and traumatology manipulative therapies. TCM physicians often prescribe CHP to treat patients with osteoporosis; however, the drugs used and their patterns of prescriptions have yet to be characterized. This study, therefore, aimed to evaluate the CHP used for the treatment of osteoporosis in Taiwan and their prescription patterns. *Methods*. A cohort of one million randomly sampled cases from the National Health Insurance Research Database (NHIRD) was analyzed to evaluate the frequencies and percentages of herbal formula and single herb prescriptions for osteoporosis. Association rules were then applied to evaluate the CHP coprescription patterns and the prevalence of osteoporosis. *Results*. The osteoporosis cohort included 16 544 patients, of whom more than 70% had used TCM on one or more occasion. Of these patients, 4 292 (25.9%) had been hospitalized at least once because of fracture. Du-Huo-Ji-Sheng-Tang and Du Zhong (Cortex Eucommiae) were the most frequently prescribed herbal formula and single herb, respectively, for the treatment of osteoporosis. *Conclusion*. This study identified patterns of CHP use for the treatment of osteoporosis. However, further research is required to fully elucidate the efficacy and safety of these CHP.

## 1. Introduction

Osteoporosis is a major public health problem and characterized by low bone mass, microarchitectural disruption, and skeletal fragility [1], resulting in increased risk of fracture and potentially increased patient morbidity [2, 3]. The prevalence of osteoporosis is higher in women than in men, and increasing prevalence is associated with increasing age in both sexes [4]. Treatment of osteoporosis consists of pharmacotherapy and lifestyle measures, including dietary changes, mineral supplementation, and exercise programs [5, 6]. Traditional Chinese medicine (TCM) is a major component of health care in Taiwan and provides one treatment option for osteoporosis.

TCM includes decoctions, acupuncture, and traumatology manipulative therapies. Chinese herbal products (CHP) represent the modern form of decoctions, with more consistent quality [7] and modern technology for patient use. In Taiwan, the National Health Insurance (NHI) program fully reimburses for single herb or herbal formula CHP. Because of their quality, convenience, and NHI reimbursement, TCM physicians currently prescribe CHP widely. TCM physicians typically prescribe one or more herbal formulae combined with several single herbs for each prescription, depending on the patient's constitution. However, the drugs used for treatment of osteoporosis and their prescription patterns have yet to be fully elucidated.

The computerized reimbursement database of the NHI, the National Health Insurance Research Database (NHIRD), stores longitudinal data on Western and Chinese medicines. It thus, provides an optimal platform for pharmacoepidemiological research on drugs used and prescription patterns in TCM. Using the NHRID, this study aimed to evaluate the CHP used for the treatment of osteoporosis in Taiwan and their prescription patterns.

## 2. Materials and Methods

### 2.1. Data Source

In March, 1995, the Taiwanese government implemented the NHI program, which provides general health insurance coverage to almost the entire Taiwanese population. In this study, a cohort of one million patients, who were beneficiaries of the NHI program from January 1 to December 31, 2005, was randomly sampled. Data were extracted from all medical records of the cohort from 1995 to 2009. Prior to 2002, 2 disease coding schemes, the International Classification of Diseases, 9th Revision, Clinical Modification (ICD-9CM), and the ICD-9 basic tabulation list, were used simultaneously for diagnoses. Data from 2003 to 2009 were, thus, used for the purposes of this study. The cohort of one million randomly sampled patients has been demonstrated to be representative of all NHI beneficiaries. Sex distribution, age distribution, and average insured payroll-related amount showed nonsignificant differences between the cohort and the entire NHIRD. The NHIRD contains medical information including data on medical care facilities and specialties, drugs, treatment and management, patient sex and date of birth, date of visit or hospitalization, transferred identification number, and diagnoses coded in the ICD-9-CM format. 

### 2.2. Study Subjects

Using the diagnostic variables in the outpatient visit database from the NHIRD, the osteoporosis cohort was selected from the outpatient department records. The ICD-9-CM codes 733.0 (osteoporosis), 733.00 (osteoporosis, unspecified), 733.01 (senile osteoporosis), 733.02 (idiopathic osteoporosis), 733.03 (disuse osteoporosis), and 733.09 (other osteoporosis) were used for identification of the cohort. All osteoporosis-related medical records during the study period were analyzed. Patients who visited the outpatient department for osteoporosis fewer than 6 times during the study period received less osteoporosis-related therapy, and had average prescription durations approximately 70% shorter, than patients who visited the outpatient department for osteoporosis 6 times or more during the study period. Despite the lack of guidelines for followup after the diagnosis of osteoporosis and treatment initiation, the followup of patient responses to therapies is recommended [8]. In this study, patients who were lost to followup and those who visited the outpatient department for osteoporosis fewer than 6 times during the study period were excluded from the analyses. Patients who visited the outpatient department for osteoporosis 6 times or more during the study period were included in the evaluations.

### 2.3. TCM

The list of reimbursed CHP was downloaded from the website of the Bureau of NHI. Corresponding drug information on a specific mixture or name was then obtained from the Committee on Chinese Medicine and Pharmacy website, including the proportions of each constituent, date and period of drug approval, drug names, and manufacturers' codes.

### 2.4. Statistical Analysis

Drug registration numbers from the Committee on Chinese Medicine and Pharmacy website were linked to the outpatient visit records of the osteoporosis cohort. The frequencies and percentages of herbal formula and single-herb prescriptions were then analyzed. Daily doses and average prescription durations were calculated for each prescription.

TCM physicians typically treat patients with one or more herbal formulae combined with several single herbs. In this study, association rules were applied to evaluate coprescription patterns of CHP for osteoporosis. The support factor (the proportion of coprescriptions of medications A and B amongst all prescriptions) was first determined. The confidence factor (the proportion of coprescriptions of medications A and B amongst all prescriptions containing medication A) was then determined. The prevalence of osteoporosis in the study cohort was also estimated. SAS version 9.1 software (SAS Institute Inc., Cary, NC, USA) was used for data linkage and descriptive statistics on patterns of drug use.

## 3. Results

Among the cohort of one million randomly sampled patients, we identified 16 544 patients who had visited the outpatient department for osteoporosis 6 times or more during the study period.  Table 1 summarizes the characteristics of the study patients. Women accounted for approximately 82% of the osteoporosis cohort. More than 70% of the osteoporosis cohort had used TCM more than once, and 4 292 (25.9%) study patients had been hospitalized at least once because of fracture, during the 7-year study period. Femoral neck fractures (ICD-9-CM 820) were the most common type of fracture in the osteoporosis cohort. As shown in Table 2, the estimated prevalence of osteoporosis in women and men was 2.7% and 0.6%, respectively, which increased with age in both sexes. The prevalence of osteoporosis was 0.2%, 5.8%, and 16.7% in women younger than 45, aged 45–65, and older than 65, respectively. These percentages were higher than those for men in the same age groups. Women older than 65 displayed the highest prevalence of osteoporosis among all patient groups.

Table 3 displays the most commonly prescribed herbal formulae and single herbs for osteoporosis, including the frequency of prescriptions, average daily doses, and average prescription durations. The most commonly prescribed herbal formula for osteoporosis was Du-Huo-Ji-Sheng-Tang (21.4%), followed by Zuo-Gui-Wan (17.4%), Shu-Jing-Huo-Xie-Tang (14.4%), Jia-Wei-Xiao-Yao-San (12.2%), and Gui-Lu-Er-Xian-Jiao (11.0%). Du Zhong (Cortex Eucommiae; 18.4%) was the most commonly prescribed single herb for patients with osteoporosis, followed by Xu Duan (Radix Dipsaci; 13.5%), Dan Shen (Radix Salvia Miltiorrhizae; 11.5%), Niu Xi (Radix Achyranthis Bidentatae; 11.1%), and Yan Hu Suo (Rhizoma Corydalis; 9.5%).

According to the association rules in Table 4, the most commonly prescribed combination of 2-herbal formulae for osteoporosis was Du-Huo-Ji-Sheng-Tang and Shu-Jing-Huo-Xie-Tang, with a support factor of 4.42%. The most commonly prescribed combination of a herbal formula and a single herb for the treatment of osteoporosis was Du-Huo-Ji-Sheng-Tang and Du Zhong, with a support factor of 4.77%, and the most commonly prescribed combination of 2-single herbs was Xu Duan and Du Zhong, with a support factor of 5.87%. Physicians frequently supplemented Du-Huo-Ji-Sheng-Tang and Zuo-Gui-Wan, the 2 most commonly prescribed herbal formulae for osteoporosis, with single herbs, such as Du Zhong and Xu Duan, or another herbal formula.

## 4. Discussion

To our knowledge, this study is the first to use a random nationwide sample to evaluate prescription patterns of CHP for osteoporosis. Ancient and modern TCM physicians have prescribed a variety of herbal formulae or single herbs to treat osteoporosis. However, prior to this study, the CHP most commonly used for the treatment of osteoporosis in clinical practice had yet to be identified. Using the NHIRD, this study analyzed the drugs used to treat osteoporosis and their prescription patterns and identified potentially effective TCM prescription patterns for osteoporosis treatment. Further clinical trials to evaluate the safety and efficacy of these TCM prescription patterns are warranted. 

TCM physicians frequently used Du-Huo-Ji-Sheng-Tang, the most commonly prescribed herbal formula for osteoporosis, to treat a combination of symptoms including knee pain, stiffness, flaccidity, and aversion to cold. According to ancient TCM literature, these symptoms indicate disharmony caused by “wind,” “cold,” and “dampness,” and Du-Huo-Ji-Sheng-Tang can improve these. A 4-week outcome study has shown its effectiveness at reducing pain and stiffness of the knee joint and improving physical function in patients with osteoarthritis [9]. The authors observed no adverse events or drug reactions during the study period [10]. Zuo-Gui-Wan is the second most commonly prescribed herbal formula for osteoporosis. According to the ancient TCM literature, Zuo-Gui-Wan can reduce dizziness, weakness, and soreness in the lumbar and knee regions. Using an animal model, Liu et al. further identified that Zuo-Gui-Wan can prevent and treat glucocorticoid-induced osteoporosis in rats [11]. 

As shown in Table 4, modern TCM physicians often use Du-Huo-Ji-Sheng-Tang and Zuo-Gui-Wan, with appropriate modifications, to treat soreness in the lower back and osteoporosis accompanied by other symptoms. However, the support factors for coprescriptions were all less than 6%, which might have caused TCM physicians to treat patients according to a syndrome differentiation theory rather than a specific disease [12]. TCM physicians also use differing coprescription patterns to treat individual patients with the same diagnosis but different conditions.

Shu-Jing-Huo-Xie-Tang, the third most commonly prescribed herbal formula for osteoporosis, can reduce blood stasis and dispel “wind dampness” in the meridian to promote blood circulation. Shu-Jing-Huo-Xie-Tang is able to relieve both arthralgia and muscle pain. In previous studies, Shu-Jing-Huo-Xie-Tang was the most commonly prescribed herbal formula for diseases of the musculoskeletal system and connective tissue in climacteric women [13] and the second most commonly used herbal formula in Taiwan during 2004 [14]. Although TCM physicians in Taiwan frequently prescribe Shu-Jing-Huo-Xie-Tang, its effectiveness and safety is uncertified based on clinical trials. 

Jia-Wei-Xiao-Yao-San, the fourth most commonly prescribed herbal formula for osteoporosis, was also the most commonly used CHP for the relief of menopausal symptoms [13]. According to previous studies, Jia-Wei-Xiao-Yao-San can be prescribed to relieve hot flushes and other menopausal symptoms, including insomnia and emotional disturbance [15, 16]. TCM physicians rarely prescribe Jia-Wei-Xiao-Yao-San in isolation for the treatment of osteoporosis, typically combining it with another herbal formula and/or single herb depending on the patient's symptoms. Women with climacteric symptoms have a higher osteoporosis incidence rate than other patient groups. Their high incidence rates might be the reason for Jia-Wei-Xiao-Yao-San being frequently prescribed CHP for osteoporosis.

As shown in Table 3, Du Zhong and Xu Duan were the 2 most frequently prescribed single herbs for osteoporosis. According to the ancient TCM literature, Du Zhong strengthens muscles and bones, and TCM physicians often prescribe it to patients with chronic pain in the lower back and knees, weakness, dizziness, and impotence. Previous studies have shown that extracts of Du Zhong can promote collagen synthesis and suppress osteoclast activity to inhibit osteolysis [17, 18]. TCM physicians have also used Xu Duan to treat patients with pain in the lower back and knee, inflammation and traumatic injuries of the leg joints and bones, and general weakness. In a previous study conducted on mice, Xu Duan increased bone density and altered bone histomorphology [19]. Ancient TCM texts recommended the prescribing of 2-single herbs as a herb pair for 2 specific purposes: to increase the efficacy of treatment and to compensate for the insufficiency of another single herb. One example of a herb pair is Du Zhong and Xu Duan. Du Zhong and Xu Duan exert similar effects in skeletal and muscular systems, and their combination might increase their efficacies. Their use as a herb pair might be the reason for Du Zhong and Xu Duan being the most frequently prescribed combination of 2-single herbs for osteoporosis. 

The proportion of a single herb in a herbal formula is changeless; therefore, it is prescribed with another single herb to alter its proportions to suit a patient's condition. Du-Huo-Ji-Sheng-Tang and Du Zhong was the most commonly prescribed combination of a herbal formula and a single herb for the treatment of osteoporosis, even though Du Zhong is one of 15 single herbs contained in Du-Huo-Ji-Sheng-Tang. 

In this study, the prevalence of osteoporosis was higher in women than in men, and increased with age. These findings corresponded with those of previous epidemiological studies [4]. However, our osteoporosis cohort included patients who had visited the outpatient department because of osteoporosis 6 times or more during the study period (from 2003 to 2009) only. The omission of other patients might, therefore, have led to the underestimation of the prevalence of osteoporosis in Taiwan. Among the patients, femoral neck fracture (ICD-9-CM 820; 8.2%) was the most common type of fracture, followed by pathologic fracture (ICD-9-CM 733.1; 6.3%), fracture of the vertebral column without spinal cord injury (ICD-9-CM 805; 5.3%), and fracture of the radius and ulna (ICD-9-CM 813; 3.8%). All osteoporotic fractures were associated with increased mortality [2, 3]. Greater attention is, therefore, needed to reduce the risk of such fractures in osteoporosis patients.

Our study suffers from 2 limitations. First, the NHI program only reimburses for CHP prescribed by TCM physicians. Our analyses did not, therefore, include CHP or decoctions purchased directly from pharmacies. This omission might have led to the underestimation of the frequency of CHP use. However, because the NHI program comprehensively covers CHP prescribed by TCM physicians, which generally cost less than CHP or decoctions sold in TCM pharmacies, this underestimation might be of small magnitude. Second, no suitable disease coding system exists for TCM. In Taiwan, TCM physicians use the ICD-9-CM alone for diagnosis. The therapeutic principle and methods of TCM are based on the results of “syndrome differentiation”; therefore, the same disease can be classified into different TCM syndrome types and treated differently, which might have caused the variations in prescription patterns for the treatment of osteoporosis patients. The development of a coding system for future TCM diagnostic classifications could increase the efficacy of TCM evaluations and greatly facilitate TCM research [20, 21].

## 5. Conclusions

This study analyzed a cohort of one million randomly sampled patients from the NHIRD from 2003 to 2009 and identified the most commonly used herbal formula and single herb for the treatment of osteoporosis in clinical practice and their prescription patterns. However, further research is needed to determine the safety and efficacy of these CHP.

## Figures and Tables

**Table 1:** Characteristics of osteoporosis population from one million random sampling cohort of the NHIR database.

Characteristics	No.	%
Age		
<45	772	4.7%
45–65	6,836	41.3%
>65	8,936	54.0%
Sex		
Female	13,560	82.0%
Male	2,984	18.0%
TCM use during study period		
Yes	12,007	72.6%
No	4,537	27.4%
Fracture during study period		
Yes	4,292	25.9%
Fracture of neck of femur (ICD-9-CM: 820)	1,355	8.2%
Pathologic fracture (ICD-9-CM: 733.1)	1,043	6.3%
Fracture of vertebral column without mention of spinal cord injury (ICD-9-CM: 805)	876	5.3%
Fracture of radius and ulna (ICD-9-CM: 813)	633	3.8%
No	12,252	74.1%

Total	16,544	100.0%

**Table 2:** Patient number and estimated prevalence of osteoporosis in Taiwan from one million random sampling cohort of the NHIR database 2003–2009.

Age/sex	All		Female		Male	
All	16,544	1.7%	13,560	2.7%	2,984	0.6%
<45	772	0.1%	571	0.2%	201	0.1%
45–65	6,836	3.3%	6,037	5.8%	799	0.8%
>65	8,936	10.7%	6,952	16.7%	1,984	4.7%

**Table 3:** Top ten herbal formulae and single herb prescribed by traditional Chinese medicine doctors for osteoporosis (*n* = 8, 369).

	Frequency of prescription *n* (%)		Average daily dose (g)	Average duration for prescription (days)
Herbal formulae				
Du-Huo-Ji-Sheng-Tang	1,792	21.4%	11.4	11.8
Zuo-Gui-Wan	1,456	17.4%	7.0	9.8
Shu-Jing-Huo-Xie-Tang	1,204	14.4%	5.0	11.3
Jia-Wei-Xiao-Yao-San	1,019	12.2%	4.1	14.1
Gui-Lu-Er-Xian-Jiao	918	11.0%	6.0	15.0
You-Gui-Wan	589	7.0%	6.0	12.5
Ji-Sheng-Shen-Qi-Wan	572	6.8%	5.3	14.6
Shao-Yao-Gan-Cao-Tang	549	6.6%	3.5	10.0
Zhi-Bo-Di-Huang-Wan	532	6.4%	7.8	14.7
Liu-Wei-Di-Huang-Wan	527	6.3%	6.4	13.6
Single herb				
Du Zhong (Cortex Eucommiae)	1,539	18.4%	1.9	12.6
Xu Duan (Radix Dipsaci)	1,128	13.5%	1.1	12.4
Dan Shen (Radix Salvia Miltiorrhizae)	966	11.5%	1.6	14.0
Niu Xi (Radix Achyranthis Bidentatae)	933	11.1%	1.3	12.2
Yan Hu Suo (Rhizoma Corydalis)	796	9.5%	1.5	11.0
Gu Sui Bu (Rhizoma Drynariae)	658	7.9%	1.3	11.4
Wei Ling Xian (Radix Clematidis)	651	7.8%	1.1	11.8
Mu Gua (Fructus Chaenomilis)	630	7.5%	1.1	12.1
Ji Xue Teng (Caulis Spatholobi)	549	6.6%	1.2	12.5
Chuan Qi (Radix Notoginseng)	494	5.9%	1.2	11.8

**Table 4:** Coprescriptions (one to one association) of single herbs and herbal formulae for osteoporosis population.

Herbal associations	Support (%)	Confidence (%)	Transaction count	Association rule
Single herb to single herb	5.87	31.95	491	Du Zhong (Cortex Eucommiae) ⇒ Xu Duan (Radix Dipsaci)
	3.94	21.47	333	Du Zhong (Cortex Eucommiae) ⇒ Niu Xi (Radix Achyranthis Bidentatae)
	3.90	21.21	326	Du Zhong (Cortex Eucommiae) ⇒ Dan Shen (Radix Salvia Miltiorrhizae)
	3.62	26.91	303	Xu Duan (Radix Dipsaci) ⇒ Gu Sui Bu (Rhizoma Drynariae)
	3.14	27.42	263	Dan Shen (Radix Salvia Miltiorrhizae) ⇒ Wei Ling Xian (Radix Clematidis)

Herbal formula to herbal formula	4.42	20.65	370	Du-huo-ji-sheng-tang ⇒ Shu-jing-huo-xie-tang
	2.86	16.41	239	Zuo-Gui-Wan ⇒ Si-Jun-Zi-Tang
	2.64	12.33	221	Du-huo-ji-sheng-tang ⇒ Gui-Lu-Er-Xian-Jiao
	2.56	14.70	214	Zuo-Gui-Wan ⇒ Jia-Wei-Xiao-Yao-San
	2.52	39.66	211	zhi-bo-di-huang-wan ⇒ Jia-Wei-Xiao-Yao-San

Herbal formula to single herb	4.77	22.27	399	Du-huo-ji-sheng-tang ⇒ Du Zhong (Cortex Eucommiae)
	4.11	19.20	344	Du-huo-ji-sheng-tang ⇒ Xu Duan (Radix Dipsaci)
	3.69	17.24	309	Du-huo-ji-sheng-tang ⇒ Niu Xi (Radix Achyranthis Bidentatae)
	3.50	20.12	293	Zuo-Gui-Wan ⇒ Yan hu suo (Rhizoma Corydalis)
	3.06	17.58	256	Zuo-Gui-Wan ⇒ Xu Duan (Radix Dipsaci)
